# Fabrication and investigation of the optoelectrical properties of MoS_2_/CdS heterojunction solar cells

**DOI:** 10.1186/1556-276X-9-662

**Published:** 2014-12-09

**Authors:** Weixia Gu, Fan Yang, Chen Wu, Yi Zhang, Miaoyuan Shi, Xiying Ma

**Affiliations:** 1 School of Mathematics and Physics, Suzhou University of Science and Technology, 1# Kerui Road, Suzhou, Jiangsu 215009, China; 2 Electricity Engineer Department, University of Liverpool, L69 7ZX Liverpool, UK

**Keywords:** Molybdenum disulfide, CdS, Solar cells, CVD, CBD, *I*-*V* behaviors

## Abstract

Molybdenum disulfide (MoS_2_)/cadmium sulfide (CdS) heterojunction solar cells were successfully synthesized via chemical bath deposition (CBD) and chemical vapor deposition (CVD). The as-grown CdS film on a fluorine tin oxide (FTO) substrate deposited by CBD is continuous and compact. The MoS_2_ film deposited by CVD is homogeneous and continuous, with a uniform color and a thickness of approximately 10 nm. The optical absorption range of the MoS_2_/CdS heterojunction covers the visible and near-infrared spectral regions of 350 to 800 nm, which is beneficial for the improvement of solar cell efficiency. Moreover, the MoS_2_/CdS solar cell exhibits good current-voltage (*I*-*V*) characteristics and pronounced photovoltaic behavior, with an open-circuit voltage of 0.66 V and a short-circuit current density of 0.227 × 10^-6^ A/cm^2^, comparable to the results obtained from other MoS_2_-based solar cells. This research is critical to investigate more efficient and stable solar cells based on graphene-like materials in the future.

## Background

Single-layer (SL) and few-layer (FL) molybdenum disulfide (MoS_2_) recently became attractive alternative semiconductor materials for next-generation nanoelectronic applications due to their large electron mobility, large bandgap [1-5], excellent stability, and the absence of dangling bonds [6]. MoS_2_ has been widely studied and applied in many areas, such as field-effect transistors [6-13], energy harvesting [14,15], optoelectronics [16-18], cocatalysts [19-21], and counter electrodes [22,23]. Moreover, single and multilayer MoS_2_ phototransistors have been demonstrated with an on/off ratio of approximately 10^3^ and a carrier mobility of 80 cm^2^/Vs [17,18], which indicates that MoS_2_ is a promising candidate for photovoltaic solar cells. Gourmelon et al. previously reported on the use of MoS_2_ in solar cells [14], but the report did not draw much interest until recently. Yu et al. reported a TiO_2_/MoS_2_/P_3_HT bulk heterojunction solar cell with a short-circuit current density of 4.7 mA/cm^2^, an open-circuit voltage of 560 mV, and a power conversion efficiency of 1.3%, as well as MoS_2_ nanomembrane-based Schottky-barrier solar cells with a power conversion efficiency of 0.7% for approximately 110-nm MoS_2_ and 1.8% for approximately 220-nm MoS_2_[24,25]. Clearly, the optical current, voltage, and energy transfer efficiency of these cells are low, and further investigations of MoS_2_-based solar cells are significant and necessary.

It is well known that cadmium sulfide (CdS), with a large direct bandgap of 2.4 eV [26-28], is a viable material and widely used in solar cells as a window layer. Zhang et al. have demonstrated MoS_2_/CdS heterojunction by photoelectrochemical methods and studied the photocatalytic and contact interface properties [15,19,29,30]. However, the photoelectric characteristics and conversion efficiency of MoS_2_/CdS heterojunction solar cells have not been demonstrated. And the complexity of these methods or the poor morphologies and structures of samples limited its use. Here, we present the fabrication of MoS_2_-based solar cells composed of p-MoS_2_ and n-CdS by simply using chemical bath deposition (CBD) and chemical vapor deposition (CVD). CBD is considered to be a low-cost and simple approach, which can produce reproducible, uniform, and adherent CdS films [31-33]. Additionally, CVD has been recognized as one of the best techniques for the fabrication of large-area homogeneous MoS_2_ films [12,13,34-36]. Moreover, we systematically analyzed the individual films' surface morphologies, structures and electrical and optical properties, as well as the photovoltaic properties of the MoS_2_/CdS films and heterojunction solar cells.

## Methods

**Figure 1:**
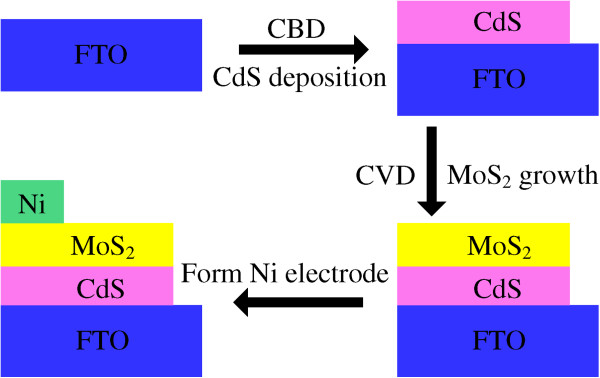
**Schematic illustration of the major steps of the fabrication of MoS**_**2**_**/CdS heterojunction solar cell.** Illustration includes CdS deposition, MoS_2_ growth, and Ni electrode deposition.

MoS_2_/CdS heterojunction solar cells were synthesized, as shown in Figure 1, in a three-step process: (i) CBD of CdS on a fluorine tin oxide (FTO)-coated glass substrate using the reaction between CdAc_2_ and H_2_NCSNH_2_, (ii) CVD of MoS_2_ on CdS, and (iii) sputtering of Ni electrodes on MoS_2_. CdS thin films were firstly deposited via CBD on FTO substrates that had been ultrasonically cleaned with deionized water, and then dried at 80°C in a drying oven. The FTO substrates were immersed in a solution composed of 0.007 M cadmium acetate (Cd(CH_3_COO)_2_ · 2H_2_O) and 0.05 M thiourea (H_2_NCSNH_2_) and maintained at 80°C for 60 min with stirring to obtain uniform deposition. After deposition, the CdS films were ultrasonically washed to remove the loosely adhered CdS particles on the surface and subsequently dried and annealed at 400°C for 60 min in N_2_ to improve the crystalline quality. Some CdS films were set aside as representative samples for characterization of surface morphologies and structures, and the others were used to synthesize MoS_2_/CdS heterojunction solar cells.

MoS_2_/CdS heterojunctions were formed by further CVD of a MoS_2_ thin film on the pre-existing CdS film. The CVD experimental setup consisted of a horizontal quartz tube furnace, an intake system, a vacuum system, and a water bath. The substrates were placed in the center of the furnace, and subsequently, the furnace was pumped down to 10^-2^ Pa and heated up to 550°C for 30 min. A mixed solution comprising 1 g analytical grade MoS_2_ micro powder, 1 g analytical grade silver nitrate (AgNO_3_) powder, and 200 mL of diluted sulfuric acid (H_2_SO_4_) was formed by stirring for 5 min and maintained at 70°C via the water bath. Ar gas was then flowed through the mixed solution with a flow rate of 20 standard cm^3^/min, carrying silver-doped MoS_2_ molecules into the furnace. The adsorption and deposition of MoS_2_ molecules onto the CdS films yielded MoS_2_/CdS thin films. After the completion of the deposition, the samples were annealed at 600°C for 30 min in an Ar atmosphere. Furthermore, to investigate the material properties of MoS_2_ films, MoS_2_ samples were deposited on quartz crystalline slides by the same method.

To construct a MoS_2_/CdS heterojunction solar cell, Ni electrodes were sputtered onto the corner of the MoS_2_/CdS thin films using magnetron sputtering. The surface morphologies and crystalline structures of MoS_2_ and CdS films were characterized using atomic force microscopy (AFM) and X-ray diffraction (XRD), respectively. The electrical properties of the samples were analyzed by a Hall Effect Measurement System (HMS-3000, Ecopia, Anyang, South Korea) at room temperature. The UV-visible absorption spectra of the samples were investigated by a UV-visible spectrophotometer (Shimadzu UV-3600, Kyoto, Japan). Photovoltaic measurements of the MoS_2_/CdS heterojunction solar cells were taken using a Keithley 4200 semiconductor characterization system (Keithley Instruments, Inc., Cleveland, OH, USA), both in the dark and under standard AM 1.5 illumination (100 mW/cm^2^).

## Results and discussion

**Figure 2:**
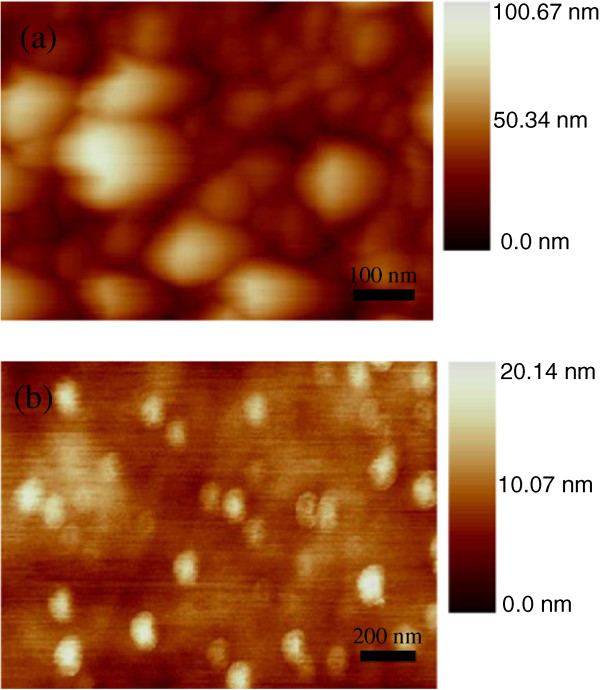
**AFM images of samples. (a)** The CdS film. **(b)** The deposited MoS_2_ film on a quartz crystalline slide.

Figure 2 shows the AFM images of the CdS film and the MoS_2_ film on a quartz crystalline slide. The surface of the CdS shown in Figure 2a is continuous and compact, and some nanoparticles are present on the top layer, which can effectively promote the absorption of light. Additionally, many MoS_2_ quantum dots around 100 nm in diameter, shown in Figure 2b, are uniformly deposited on the surface of the MoS_2_ film. Under the quantum dots, the MoS_2_ film is homogeneous and continuous, with a uniform color and a thickness of about 10 nm, which is equal to a few layers of MoS_2_. This growth mode, called the layer-quantum dot mode, corresponds to the hexagonal crystalline structure of MoS_2_.

**Figure 3:**
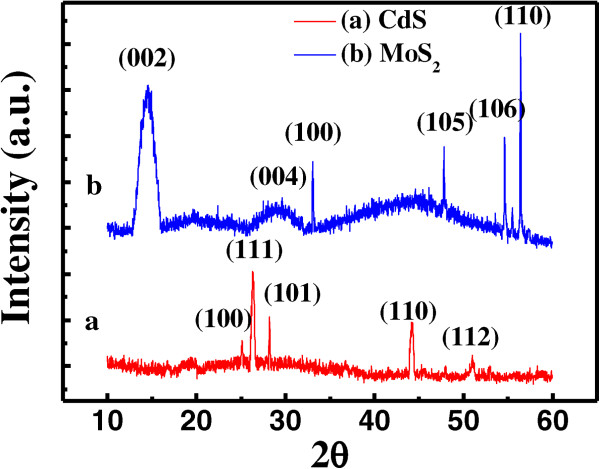
**XRD patterns of the CdS and MoS**_**2 **_**films for the diffraction angle in the range of 10° ~ 60°.**

The crystal structures of the samples were characterized by XRD. The XRD pattern of the CdS film is illustrated in Figure 3a. Only the (111) diffraction peak, appearing at 26.2°, belongs to cubic CdS; the others, located at 24.8°, 28.2°, 43.7°, and 50.8°, correspond to the (100), (101), (110), and (112) diffraction planes of a hexagonal CdS, respectively, which is more suitable to be an n-type window layer for solar cells, due to its high transmission and electrical conductivity [37]. Moreover, these observed diffraction peaks are rather sharp, especially the (111) and (101) peaks, which indicate good crystallinity. Figure 3b shows the XRD pattern of the MoS_2_ film. Six sharp diffraction peaks are located at 14.7°, 29.3°, 33.1°, 47.8°, 54.6°, and 56.4°, corresponding to the (002), (004), (100), (105), (106), and (110) crystal planes of MoS_2_, respectively, which show that the MoS_2_ film exhibits a variety of crystal structures. In addition, it has to be noted that no silver diffraction peaks are observed, indicating that the silver doping does not change the crystal structure of the MoS_2_ film.

**Figure 4:**
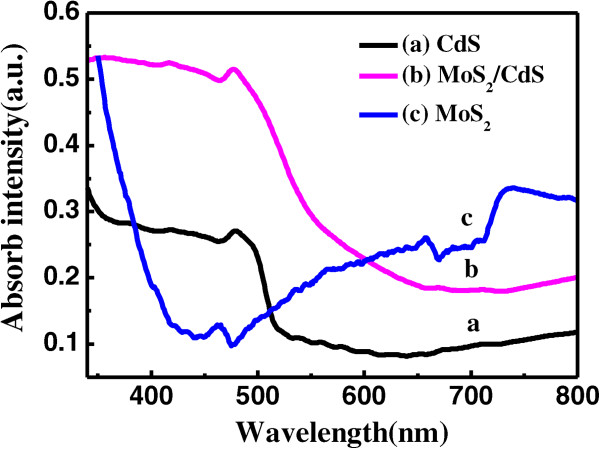
**UV-vis absorption spectra of MoS**_**2**_**, CdS, and MoS**_**2**_**/CdS samples in the wavelength region of 350 to 800 nm.**

Figure 4 shows the ultraviolet-visible (UV-vis) absorption spectra of the CdS, MoS_2_, and MoS_2_/CdS samples in the wavelength region of 350 to 800 nm. The CdS film has a strong optical absorption peak at 490 nm, and the optical absorption covers the wavelength region of 350 to 510 nm, consistent with the previously reported findings [29,38]. Over the region 510 to 800 nm, the absorptivity of the CdS film decreases abruptly, and no other absorption peaks are observed, indicating that the CdS film is transparent to light in this range. However, there is an absorption peak observed for the MoS_2_ film located at 735 nm, which corresponds to the MoS_2_ bandgap of about 1.69 eV. The optical absorption range of the MoS_2_ film almost covers the range that the CdS film does not absorb light, demonstrating that MoS_2_/CdS solar cells enhance the absorption of light, compared with silicon-based solar cells. Moreover, the optical absorption range of the MoS_2_/CdS sample covers the visible and near-infrared spectral regions of 350 to 800 nm, which is beneficial for the improvement of solar cell efficiency.

**Figure 5:**
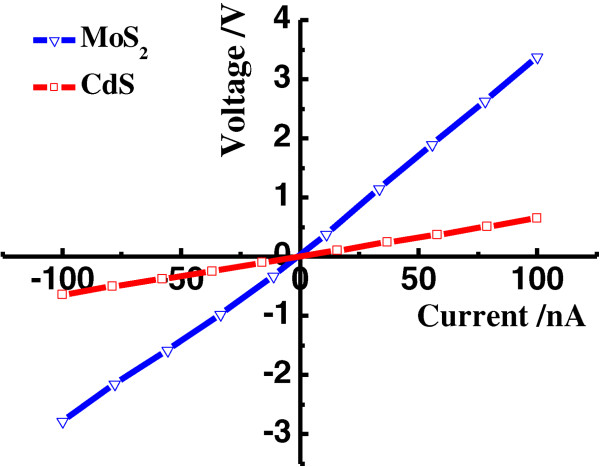
**The surface current-voltage ( *I *- *V *) curves of the two measured points on the CdS and MoS**_**2 **_**films.**

We measured surface current-voltage (*I*-*V*) properties, carrier mobilities, and Hall coefficients of the MoS_2_ and CdS samples using a Hall Effect measurement system. Figure 5 shows the surface *I*-*V* behaviors of the two measured points on the samples. The extracted voltages between the two points show a linear dependency on the applied current, indicating that the MoS_2_ and CdS films have good conductivity, with few surface defects or impurities. The electron mobilities in the MoS_2_ and CdS films are 1.579 × 10^3^ cm^2^/Vs and 7.68 × 10^2^ cm^2^/Vs, respectively. Note that the mobility value for the MoS_2_ film is higher than previously reported [39,40], which may be due to lower phonon and lattice scattering. Furthermore, the Hall coefficients of the MoS_2_ and CdS films are 6.379 × 10^6^ cm^3^/C and -3.257 × 10^2^ cm^3^/C, respectively, showing that MoS_2_ is a p-type semiconductor, and it can form a p-n junction with n-type CdS, as demonstrated in previous studies [15,19,29,41-43].

**Figure 6:**
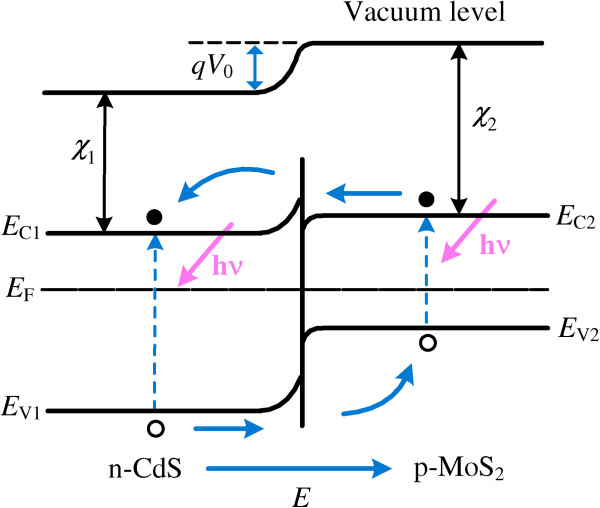
**The energy band diagram of the fabricated MoS**_**2**_**/CdS heterojunction solar cell upon light illumination.**

Figure 6 displays the energy band diagram of the fabricated MoS_2_/CdS heterojunction solar cell. *E*_C1_, *E*_C2_, *E*_V1_, and *E*_V2_ denote the conduction bands and valence bands of CdS and MoS_2_, respectively. *E*_F_ is the Fermi level energy. *χ*_1_ and *χ*_2_ are the electron affinities of CdS (3.8 eV) [38] and MoS_2_ (4.0 eV), respectively. *V*_0_ is the built-in potential, and *E*, with the direction from n-CdS to p-MoS_2,_ is the built-in electric field. Because of the Fermi level difference between n-CdS and p-MoS_2_, electrons diffuse from n-CdS to p-MoS_2_, and simultaneously, holes in p-MoS_2_ move to n-CdS, leading to the formation of a space-charge region and built-in electric field with the direction from n-CdS to p-MoS_2_ at the contact interface. The built-in electric field, *E*, prevents carriers from diffusing and makes them drift in the opposite direction, and finally, the heterojunction comes to thermal equilibrium with a unified Fermi level. Under light illumination, the photogenerated electrons and holes are quickly separated and driven into n-CdS and p-MoS_2_, respectively, under the acceleration of *E*, which gives rise to the generation of the photocurrent.

**Figure 7:**
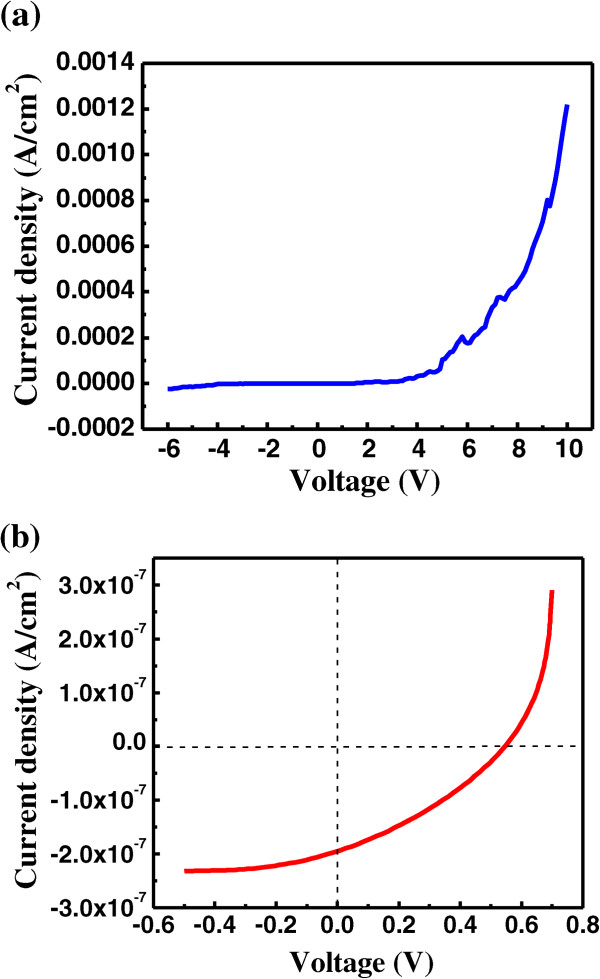
**The *J*-*V *behaviors of MoS**_**2**_**/CdS heterojunction solar cells. (a)** Dark *J*-*V* characteristics of MoS_2_/CdS heterojunction solar cell. **(b)** Illuminated *J*-*V* characteristics of MoS_2_/CdS heterojunction solar cell.

Figure 7a shows the dark current density-voltage (*J*-*V*) characteristics of the fabricated MoS_2_/CdS heterojunction solar cell. Remarkably, the current curve of the device shows an exponential dependence on the applied positive voltage, and tends to be almost zero under the reverse voltage, indicating that the MoS_2_/CdS solar cell exhibits good rectification characteristics, and forms a well-defined p-n junction, as demonstrated by the previous reports [15,19,29].

Figure 7b displays the light-illuminated *J*-*V* characteristics of the fabricated MoS_2_/CdS heterojunction solar cell. The solar cell exhibits pronounced photovoltaic behavior, with an open-circuit voltage (*V*_oc_) of 0.66 V and a short-circuit current density (*J*_sc_) of 0.227 × 10^-6^ A/cm^2^. We can see that *V*_oc_ is much larger than the results obtained from other MoS_2_-based solar cells [24,25], but *J*_sc_ is much lower than that of common solar cells [24,25], which is likely attributed to the large resistances for the device. The fill factor (FF) can be obtained based on the relationship of FF = *J*_m_*V*_m_/*J*_sc_*V*_oc_, where *J*_m_ and *V*_m_ are the current density and voltage at the maximum power output, respectively. In this instance, FF is approximately 0.22, comparable to previously reported values [25]. These results show that to improve the light energy efficiency of the MoS_2_/CdS heterojunction solar cells it is necessary to lower the contact resistance of the cell, which is also critical to solar cells based on graphene-like materials.

## Conclusions

We have fabricated heterojunction solar cells composed of p-MoS_2_ and n-CdS films using CBD and CVD methods and studied the surface morphologies, structures, and electrical and optical properties, as well as the photovoltaic properties. The MoS_2_ film is homogeneous and continuous, with a thickness of around 10 nm, which is equal to a few layers of MoS_2_. The as-grown CdS film is continuous and compact. The optical absorption range of the MoS_2_/CdS film covers the visible and near-infrared spectral regions of 350 to 800 nm, which is beneficial for improving solar cell efficiency. Moreover, the MoS_2_/CdS solar cell exhibits good rectification characteristics and pronounced photovoltaic behavior, with a short-circuit current density of 0.227 × 10^-6^ A/cm^2^ and an open-circuit voltage of 0.66 V, comparable to the results obtained from other MoS_2_-based solar cells.

## Competing interests

The authors declare that they have no competing interests.

## Authors' contributions

WG participated in the fabrication of MoS_2_/CdS heterojunction solar cells, measured the electrical properties of the solar cells, and wrote the manuscript. FY, CW, YZ, and MS investigated the surface morphologies, structures, and electrical and optical properties of the samples and participated in the analysis of the results of the solar cells. XM designed the structure of the solar cell and analyzed the results. All authors read and approved the final manuscript.

## Authors' information

WG is a graduate student major in fabrication of new semiconductor nanometer materials. FY, CW, YZ, and MS are undergraduates. XM is a professor and PhD-degree holder specializing in semiconductor materials and devices, especially expert in nanoscaled optical-electronic materials and optoelectronic devices.

## References

[B1] Kam KK Parkinson BA Detailed photocurrent spectroscopy of the semiconducting group VIB transition metal dichalcogenides J Phys Chem 1982 86 463 467 10.1021/j100393a010

[B2] Lebègue S Eriksson O Electronic structure of two-dimensional crystals from *ab initio* theory Phys Rev B 2009 79 115409 115412

[B3] Splendiani A Sun L Zhang Y Li T Kim J Chim CY Galli G Wang F Emerging photoluminescence in monolayer MoS_2_ Nano Lett 2010 10 1271 1275 10.1021/nl903868w 20229981

[B4] Mak KF Lee C Hone J Shan J Heinz TF Atomically thin MoS_2_: a new direct-gap semiconductor Phys Rev Lett 2010 105 136805 21230799 10.1103/PhysRevLett.105.136805

[B5] Kuc A Zibouche N Heine T Influence of quantum confinement on the electronic structure of the transition metal sulfide TS_2_ Phys Rev B 2011 83 245213 245216

[B6] Radisavljevic B Radenovic A Brivio J Giacometti V Kis A Single-layer MoS_2_ transistors Nat Nanotechnol 2011 6 147 150 10.1038/nnano.2010.279 21278752

[B7] Radisavljevic B Whitwick MB Kis A Integrated circuits and logic operations based on single-layer MoS_2_ ACS Nano 2011 5 9934 9938 10.1021/nn203715c 22073905

[B8] Qiu H Pan L Yao Z Li J Shi Y Wang X Electrical characterization of back-gated bi-layer MoS_2_ field-effect transistors and the effect of ambient on their performances Appl Phys Lett 2012 100 123104 10.1063/1.3696045

[B9] Das S Chen HY Penumatcha AV Appenzeller J High performance multilayer MoS_2_ transistors with scandium contacts Nano Lett 2013 13 100 105 10.1021/nl303583v 23240655

[B10] Yoon Y Ganapathi K Salahuddin S How good can monolayer MoS_2_ transistors be? Nano Lett 2011 11 3768 3773 10.1021/nl2018178 21790188

[B11] Kim S Konar A Hwang WS Lee JH Lee J Yang J Jung C Kim H Yoo JB Choi JY Jin YW Lee SY Jena D Choi W Kim K High-mobility and low-power thin-film transistors based on multilayer MoS_2_ crystals Nat Commun 2012 3 1011 22910357 10.1038/ncomms2018

[B12] Liu KK Zhang W Lee YH Lin YC Chang MT Su CY Chang CS Li H Shi Y Zhang H Lai CS Li LJ Growth of large-area and highly crystalline MoS_2_ thin layers on insulating substrates Nano Lett 2012 12 1538 1544 10.1021/nl2043612 22369470

[B13] Gu W Shen J Ma X Fabrication and electrical properties of MoS_2_ nanodisc-based back-gated field effect transistors Nanoscale Res Lett 2014 9 100 10.1186/1556-276X-9-100 24576344 PMC3943990

[B14] Gourmelon E Lignier O Hadouda H Couturier G Bernède JC Tedd J Pouzet J Salardenne J MS_2_ (M = W, Mo) photosensitive thin films for solar cells Sol Energy Mater Sol Cells 1997 46 115 121 10.1016/S0927-0248(96)00096-7

[B15] Zong X Yan H Wu G Ma G Wen F Wang L Li C Enhancement of photocatalytic H_2_ evolution on CdS by loading MoS_2_ as cocatalyst under visible light irradiation J Am Chem Soc 2008 130 7176 7177 10.1021/ja8007825 18473462

[B16] Takahashi T Takenobu T Takeya J Iwasa Y Ambipolar light-emitting transistors of a tetracene single crystal Adv Funct Mater 2007 17 1623 1628 10.1002/adfm.200700046

[B17] Yin Z Li H Li H Jiang L Shi Y Sun Y Lu G Zhang Q Chen X Zhang H Single-layer MoS_2_ phototransistors ACS Nano 2012 6 74 80 10.1021/nn2024557 22165908

[B18] Lee HS Min SW Chang YG Park MK Nam T Kim H Kim JH Ryu S Im S MoS_2_ nanosheet phototransistors with thickness-modulated optical energy gap Nano Lett 2012 12 3695 3700 10.1021/nl301485q 22681413

[B19] Zong X Wu G Yan H Ma G Shi J Wen F Wang L Li C Photocatalytic H_2_ evolution on MoS_2_/CdS catalysts under visible light irradiation J Phys Chem C 2010 114 1963 1968 10.1021/jp904350e

[B20] Yang L Zhong D Zhang J Yan Z Ge S Du P Jiang J Sun D Wu X Fan Z Dayeh SA Xiang B Optical properties of metal-molybdenum disulfide hybrid nanosheets and their application for enhanced photocatalytic hydrogen evolution ACS Nano 2014 8 6979 6985 10.1021/nn501807y 24884001

[B21] Chang K Mei Z Wang T Kang Q Ouyang S Ye J MoS_2_/graphene cocatalyst for efficient photocatalytic H_2_ evolution under visible light irradiation ACS Nano 2014 8 7078 7087 10.1021/nn5019945 24923678

[B22] Freitas FS Gonçalves AS Morais AD Benedetti JE Nogueira AF Graphene-like MoS_2_ as a low-cost counter electrode material for dye-sensitized solar cells NanoGe J Ener Sust 2013 1 011002

[B23] Wu M Wang Y Lin X Yu N Wang L Wang L Hagfeldt A Ma T Economical and effective sulfide catalysts for dye-sensitized solar cells as counter electrodes Phys Chem Chem Phys 2011 13 19298 19301 10.1039/c1cp22819f 21984309

[B24] Shanmugam M Bansal T Durcan CA Yu B Molybdenum disulphide/titanium dioxide nanocomposite-poly 3-hexylthiophene bulk heterojunction solar cell Appl Phys Lett 2012 100 153901 10.1063/1.3703602

[B25] Shanmugam M Durcan CA Yu B Layered semiconductor molybdenum disulfide nanomembrane based Schottky-barrier solar cells Nanoscale 2012 4 7399 7405 10.1039/c2nr32394j 23085834

[B26] Kamat PV Meeting the clean energy demand: nanostructure architectures for solar energy conversion J Phys Chem C 2007 111 2834 2860 10.1021/jp066952u

[B27] Kalyanasundaram K Grätzel M Pelizzetti E Interfacial electron transfer in colloidal metal and semiconductor dispersions and photodecomposition of water Coord Chem Rev 1986 69 57 125 10.1016/0010-8545(86)85009-3

[B28] Ashokkumar M An overview on semiconductor particulate systems for photoproduction of hydrogen Int J Hydrogen Energy 1998 23 427 438 10.1016/S0360-3199(97)00103-1

[B29] Liu Y Yu YX Zhang WD MoS_2_/CdS heterojunction with high photoelectrochemical activity for H_2_ evolution under visible light: the role of MoS_2_ J Phys Chem C 2013 117 12949 12957 10.1021/jp4009652

[B30] Lee JK Lee W Yoon TJ Park GS Choy JH A novel quantum dot pillared layered transition metal sulfide: CdS-MoS_2_ semiconductor-metal nanohybrid J Mater Chem 2002 12 614 618 10.1039/b108062h

[B31] Zinoviev KV Zeleya-Angel O Influence of low temperature thermal annealing on the dark resistivity of chemical bath deposited CdS films Mater Chem Phys 2001 70 100 102 10.1016/S0254-0584(00)00377-1

[B32] Mahanty S Basak D Rueda F Leon M Optical properties of chemical bath deposited CdS thin films J Electron Mater 1999 28 559 562 10.1007/s11664-999-0112-0

[B33] Dzhafarov TD Altunbas M Kopya AI Novruzov V Bacaksiz E Formation of p-type CdS thin films by laser-stimulated copper diffusion J Phys D Appl Phys 1999 32 L125 L128 10.1088/0022-3727/32/24/101

[B34] Zhan Y Liu Z Najmaei S Ajayan PM Lou J Large-area vapor-phase growth and characterization of MoS_2_ atomic layers on a SiO_2_ substrate Small 2012 8 966 971 10.1002/smll.201102654 22334392

[B35] Liu H Si M Najmaei S Neal AT Du Y Ajayan PM Lou J Ye PD Statistical study of deep submicron dual-gated field-effect transistors on monolayer chemical vapor deposition molybdenum disulfide films Nano Lett 2013 13 2640 2646 10.1021/nl400778q 23679044

[B36] Lee YH Zhang XQ Zhang W Chang MT Lin CT Chang KD Yu YC Wang JTW Chang CS Li LJ Lin TW Synthesis of large-area MoS_2_ atomic layers with chemical vapor deposition Adv Mater 2012 24 2320 2325 10.1002/adma.201104798 22467187

[B37] Yeh CY Lu ZW Froyen S Zunger A Zinc-blende-wurtzite polytypism in semiconductors Phys Rev B 1992 46 10086 10097 10.1103/PhysRevB.46.10086 10002848

[B38] Jia L Wang DH Huang YX Xu AW Yu HQ Highly durable N-doped graphene/CdS nanocomposites with enhanced photocatalytic hydrogen evolution from water under visible light irradiation J Phys Chem C 2011 115 11466 11473

[B39] Novoselov KS Jiang D Schedin F Booth TJ Khotkevich VV Morozov SV Geim AK Two-dimensional atomic crystals Proc Natl Acad Sci U S A 2005 102 10451 10453 10.1073/pnas.0502848102 16027370 PMC1180777

[B40] Ayari A Cobas E Ogundadegbe O Fuhrer MS Realization and electrical characterization of ultrathin crystals of layered transition-metal dichalcogenides J Appl Phys 2007 101 014507 10.1063/1.2407388

[B41] Heising J Kanatzidis MG Exfoliated and restacked MoS_2_ and WS_2_: ionic or neutral species? Encapsulation and ordering of hard electropositive cations J Am Chem Soc 1999 121 11720 11732 10.1021/ja991644d

[B42] Gourmelon E Bernede JC Pouzet J Marsillac S Textured MoS_2_ thin films obtained on tungsten: electrical properties of the W/MoS_2_ contact J Appl Phys 2000 87 1182 1186 10.1063/1.372061

[B43] He J Wu K Sa R Li Q Wei Y Magnetic properties of nonmetal atoms absorbed MoS_2_ monolayers Appl Phys Lett 2010 96 082504 10.1063/1.3318254

